# The Hospital World

**Published:** 1910-06-04

**Authors:** 


### The Hospital World.

### Personalia.

Mr. P. W. Rowley, who for nearly seven years has occupied the post of steward's second clerk at the St. Mary, Islington, Poor-Law Infirmary, has been presented by the officers of the institution with a kit-bag on his leaving to take up the post of steward's clerk at the Leicester Poor-Law Infirmary.

Sir William Collins has been re-elected President of the Asylum Workers' Association, and an illuminated address was presented to him in grateful recognition of his successful efforts in the passing of the Asylum Officers' Superannuation Act. Another worker, Dr. Shuttleworth, was presented with a Remington typewriter and a pocket-book containing a cheque in appreciation of his labours in the promotion of the Bill.

Sir Horace Brooks Marshall has been unanimously elected a Vice-President of the Royal Hospital for Incurables, Putney Heath. Sir Horace was to have taken the chair at the festival dinner of the Institution, which was cancelled in consequence of the death of King Edward. Despite the cancelling of the dinner, four days before it would have taken place the chairman's list amounted to over £4,000.

The League of Mercy has several hundreds of thousands of associates, in addition to members, vice-presidents, lady presidents, and presidents of districts. One of these associates was an old servant, a Mrs. M. E. Warman, who has recently died and bequeathed some of her property to be devoted to charities. Her former mistress, as executrix to the will, has given £100 of this money to the League of Mercy, to which the late Mrs. Warman subscribed regularly 1s. annually during her lifetime. This is the first legacy of the kind which the League has received, and it indicates the importance and value of enlisting the sympathies of small subscribers to the voluntary hospitals.

Before proceeding to the business at the meeting of the Longtown (Cumberland) Guardians, at the last meeting, the Chairman referred to the death of Mr. C. B. Hodgson, the late clerk of the Board, whose name is well known in connection with institutional and charitable work in the district. Mr. Hodgson was clerk of the Board for sixty years. A vote of condolence with his family was passed in silence. Mr. Hodgson was one of the best-known figures in the North of England. Born in the reign of George IV. in 1824, he lived till the morning when George V. was proclaimed King in London. Having qualified as a solicitor in 1846, he had completed sixty-four years of professional work, though for some months past increasing infirmity had confined him to his home at Harker Grange, near Carlisle. For sixty years he served as clerk to the Longtown Union, and for forty-seven years he was clerk to the Longtown Magistrates, while last year he resigned the clerkship to the Cumberland Ward Magistrates after fifty-seven years' service. In 1891 he succeeded his late brother, Mr. T. H. Hodgson, as clerk of the peace for Cumberland and clerk to the County Council, thus occupying as clerk of the peace a position formerly held by his father, Mr. William Hodgson. In private life he was widely known for his generosity. For sixty-four years he carried on Blackford Sunday-school, near his home, as superintendent, and for half a century he was churchwarden at Houghton. He was also interested in foreign missions, being local treasurer and a life governor of the British and Foreign Bible Society. His only son, Mr. C. W. Allan Hodgson, has succeeded him as clerk to the Longtown Union, clerk to the Lunacy Committee, to the Cumberland Ward Magistrates, and other offices.

### Elections and Appointments.

Captain Webbe, a very old friend to St. Mary's Hospital, has been elected a Vice-President.

Mrs. Blencoe Cookson has been re-elected President of the Morpeth Cottage Hospital.

At a meeting of the Special Committee for the election of honorary medical officers of the Manchester Royal Infirmary, held on May 24, Mr. Chas. Roberts, M.B., B.S. (Lond.), F.R.C.S. (Eng.), L.R.C.P. (Lond.), was appointed an honorary assistant surgeon of the institution to fill the vacancy caused by the promotion of Mr. J. W. Smith, F.R.C.S. (Eng.), to be an honorary surgeon on the retirement of Mr. F. A. Southam, F.R.C.S. (Eng.).

Mr. Arthur Heywood has been elected Chairman of the Board of Governors of the Manchester Children's Hospital, Pendlebury, and Mr. William Buckley, J.P., has been elected Vice-Chairman of the Board and Chairman of the House Committee.

The following gentlemen have been elected as the Management Committee of the Victoria Jubilee Cottage Hospital, Watton, Norfolk : The Rev. C. B. Nash, the Rev. H. B. Upcher, the Hon. C. B. Hanbury, the Rev. J. H. Rose, the Rev. J. Thomas, with Dr. T. A. Alexander, Dr. H. Mallins and Dr. J. Panting, and Mr. Charles Robinson (Hon. Secretary), as ex-officio members.

Mr. T. R. Ferens, M.P., has been elected President of the Hull Victoria Hospital for Sick Children for the coming year.

Lady Wentworth has become a Patroness of the National Anti-Vivisection Hospital, and Sir Capel C. Wolseley has joined the board of management.

The Duke of Fife has been re-elected president of the Hospital for Sick Children, Great Ormond Street.

The Rev. F. B. Johnston, the Rev. E. A. Saunders, Mr. J. P. Bevan, Mr. W. Donston, Mr. F. Jenkins, Mr. H. Bryant, and Mr. W. D. Cornish, retiring governors of the Prince of Wales' General Hospital, Tottenham, have been re-elected.

